# Serum interleukin‐33 as a novel marker for long‐term prognosis and recurrence in acute ischemic stroke patients

**DOI:** 10.1002/brb3.1369

**Published:** 2019-08-09

**Authors:** Xian‐Mei Li, Xiao‐yang Wang, Xiao‐Wen Feng, Meng‐Meng Shao, Wen‐Fang Liu, Qin‐Qin Ma, En‐Pei Wang, Jie Chen, Bei Shao

**Affiliations:** ^1^ Department of Rehabilitation First Affiliated Hospital of Wenzhou Medical University Wenzhou China; ^2^ Department of Neurology First Affiliated Hospital of Wenzhou Medical University Wenzhou China

**Keywords:** acute ischemic stroke, inflammation, interleukin‐33, long‐term functional outcome, recurrence

## Abstract

**Objectives:**

Interleukin‐33, a newly identified member of interleukin‐1 family, had been confirmed to play a crucial role in regulating inflammatory responses in various disease. However, the exact role of interleukin‐33 in the disease process of acute ischemic stroke still remains unclear. This study aims to demonstrate the relationship between interleukin‐33 levels and long‐term functional outcome as well as ischemic stroke recurrence.

**Methods:**

Three hundred and four first‐ever acute ischemic stroke patients were recruited and basic information and history of all subjects taken within 72 hr on admission. The functional outcome was estimated by Barthel index. The multivariate logistic regression was used to analyze the prognosis, while the Cox proportional hazard model was applied to assess the recurrence risk.

**Results:**

Out of 304 subjects, 259 patients successfully completed scheduled two‐year follow‐up. We found that higher interleukin‐33 levels correlated positively with better prognosis as compared with those with lower interleukin‐33 levels who presented with poorer outcome (62.45 ± 20.50 ng/ml vs. 51.58 ± 19.16 ng/ml, *p* < .001). After adjustment of all confounders, interleukin‐33 was associated with the one‐year prognosis with an adjusted odds ratio of 0.956 (95% confidence interval, 0.937–0.976, *p* < .001). Furthermore, interleukin‐33 levels were also closely related to recurrent ischemic stroke with an adjusted hazard ratio of 0.979 (95% confidence interval, 0.961–0.997, *p* = .025).

**Conclusions:**

IL‐33 can be used to predict the long‐term outcomes and ischemic stroke recurrence in first‐ever acute ischemic stroke patients.

## 1 INTRODUCTION

Stroke is the second major leading cause of mortality and long‐term functional disability behind ischemic heart disease. The incidence of stroke‐related disability and dependence is estimated to increase in the next 10 years due to increase in aging population (Donnan, Fisher, Macleod, & Davis, 2008). Ischemic stroke which results mainly from the blockage of cerebral blood flow has been seen to account for about 87% of all the cerebrovascular accidents (Liu, Wang, Wong, & Wang, 2011). Although many studies on stroke have been done and some still ongoing, there still remains an available biomarker which enables clinicians to readily evaluate this condition and to accurately predict the outcome of this disease.

Many studies had highlighted inflammatory cascade to play a pivotal role in the development and progression of cerebral ischemia especially at the acute stage (Amantea et al., 2014; Cotrina, Lou, Tome‐Garcia, Goldman, & Nedergaard, 2017). Inflammation has however been seen to have dual functions in AIS, that is, either by increasing tissue damage through the release of cytotoxic cytokines or by the release of anti‐inflammatory cytokines which serves neuroprotective functions (Danton & Dietrich, 2003; McCombe & Read, 2008). Emerging findings have therefore suggested the use of inflammatory‐mediated pathways as targets in the treatment of cerebrovascular diseases. For example, T cells (including Th1‐type and Th2‐type) and their subsets are considered crucial in pathogenesis of inflammation‐mediated cerebrovascular disease and as such could be used as therapeutic targets in stroke patients (Arumugam, Granger, & Mattson, 2005). By inducing the polarization of macrophage into M1‐type and causing many kinds of pro‐inflammatory cytokines, Th1‐type response promotes inflammation leading to further secondary brain injury. However, Th2‐type response produces anti‐inflammatory cytokine though induces macrophage polarization into M2‐type which gives neuroprotective effects (Gee, Kalil, Shea, & Becker, 2007). Thus, the balances between Th1/Th2 type response might serve as a key point to regulate the inflammatory response. Interleukin‐33 (IL‐33), a novel member of the interleukin‐1 (IL‐1) family, is closely related to regulation of inflammatory responses in central nervous system (Schmitz et al., 2005). IL‐33 functions as traditional cytokine which is released from necrotic cells signaling or “alarming” of impending tissue injury. Another feature of IL‐33 identified in various studies is its role in gene transcription. IL‐33 is considered as an intracellular nuclear factor which combines with chromosomes and regulates gene transcription (Miller, 2011). By the interaction of IL‐33 with its receptor ST2, IL‐33 can tilt the Th1/Th2 balance toward Th2 cells which could serve various protective functions in Th1‐mediated inflammatory diseases (Duan et al., 2012). It has been confirmed that serum IL‐33 was involved in various diseases including asthma, obesity, atherosclerosis, and Alzheimer disease (Fu et al., 2016; Lloyd, 2010; Miller et al., 2008, 2010).

However, the effects of IL‐33 levels on long‐term functional outcome and recurrent ischemic stroke in AIS patients are still uncertain. In this study, we aimed to investigate whether serum IL‐33 levels could be a potential biomarker for prediction of the long‐term prognosis and the recurrence of AIS.

## 2 MATERIALS AND METHODS

### 2.1 Study population

This study was approved by the Medical Ethics Committee of Wenzhou Medical University and with obtained informed consents from all participants or their legal representatives. A prospective study was carried out by the First Affiliated Hospital of Wenzhou Medical University recruiting 304 patients diagnosed with first‐ever AIS. The diagnosis of AIS was based on the duration of stroke symptoms, that is, within 3 days and according to the World Health Organization criteria for the diagnosis of stroke (Hatano, 1976. All patients with the following conditions were excluded: (a) patients with mental or physical illnesses such as hydrocephalus, dementia, traumatic brain injury, Parkinson disease, or schizophrenia; (b) patients with active infection, hematological disease, or peripheral vascular disease; (c) patients with history of cancer, liver or renal insufficiency, autoimmune diseases; (d) participants with concomitant illnesses so severe to prevent them from regular follow‐up.

### 2.2 Clinical data

The baseline clinical information covered demographic characteristics (age, gender, body mass index [BMI]) and vascular risk factors (hyperlipidemia, coronary artery disease [CAD], hypertension, type 2 diabetes mellitus, current smoking and alcohol abuse). History was taken on admission for all participants. The volume of infarct on diffusion‐weighted imaging was calculated using the formula 0.5 × *a* × *b* × c (Sims et al., 2009), with infarction considered small if infarct volume is less than 5 cm^3^. The National Institutes of Health Stroke Scale (NIHSS; Brott et al., 1989) was applied to assess stroke severity at admission and at discharge, while the Barthel Index (BI; Mahoney & Barthel, 1965) was used to measure stroke functional outcomes at one year after admission. The BI score below 90 was defined as unfavorable outcome. Telephone interviews or electronic medical records were applied to verify whether the ischemic stroke relapsed. The follow‐up period was considered as the admission time to December 31, 2016, or to the time of stroke recurrence.

Venous blood samples were acquired within 24 hr after admission. Laboratory parameters including serum biochemical indexes (total WBCs, neutrophils, alanine aminotransferase [ALT], aspartate transaminase [AST], total cholesterol [TC], triglyceride [TG], low‐density lipoprotein cholesterol [LDL‐C], glycosylated hemoglobin [HbA1c], Albumin, serum creatinine, blood urea nitrogen, uric acid, high‐sensitivity C‐reactive protein [Hs‐CRP], and IL‐33) were also quantified in hospital's clinical laboratory.

### 2.3 Statistical analysis

All statistical analyses were done using SPSS Statistics 23.0 software (SPSS Inc.). The 2‐sided *p* value of <.05 was referred to be statistically significant. According to the distribution of data, the normal continuous variables analyzed using the unpaired *t* test or one‐way analysis of variance (ANOVA) were expressed as the mean value ± standard deviation (*SD*), whereas the asymmetrically distributed variables assessed using the Kruskal–Wallis and Mann–Whitney U tests were presented as median with interquartile range (IQR). Categorical variables expressed as frequency and percentage were appropriately compared using the chi‐square test. The multivariate logistic regression analysis adjusted for the confounders in the univariate analyses was employed to verify the relationship between IL‐33 and the functional prognosis of AIS. Adjusted odds ratio (OR) with the corresponding 95% confidence interval (CI) represented the results. Furthermore, receiver operating characteristic (ROC) curves were applied to assess the accuracy of the IL‐33 to predict AIS long‐term functional outcomes. Meanwhile, area under the curve (AUC) was calculated to measure the accuracy of the test. In addition, Cox proportional hazard models were employed to evaluate the connection between IL‐33 and recurrent ischemic stroke.

## 3 RESULTS

### 3.1 Baseline characteristics

In this study, a total of 304 AIS patients met the inclusion criteria; however, 259 patients finished the two‐year follow‐up (45 patients lost contact). The average age of AIS patients was 63.14 ± 10.02 years with 170 (65.6%) of patients being male. The median NIHSS score on admission was 4.00 ((IQR, 2.00–6.00), while the median NIHSS score at discharge descended to 3.00 (IQR, 1.00–5.00). A poor functional outcome was recorded in 88 patients (33.98%; Table 1). Our results indicated a negative correlation between IL‐33 levels and HbA1c (*r* = −.175, *p* = .005), but with slight correlation with total WBCs (*r* = −.124, *p* = .046). There was also slight negative association between IL‐33 and Hs‐CRP though insignificant (*r* = −.105, *p* = .091). However, the concentrations of IL‐33 had no statistical correlation with cerebral infarction volume (58.73 ± 21.08 ng/ml vs. 58.86 ± 19.19 ng/ml, *p* > .05).

**Table 1:** Baseline characteristics of AIS patients with favorable or unfavorable outcomes

Characteristics	Total (*N* = 259)	The prognosis of 1 year		*p* Value
		Favorable outcome (*N* = 171)	Unfavorable outcome (*N* = 88)	
Age (years)	63.14 ± 10.02	62.22 ± 22.19	64.93 ± 9.98	.039
Male no. (%)	170 (65.6)	114 (66.7)	56 (63.6)	NS
SBP (mmHg)	161.22 ± 22.55	160.29 ± 22.19	163.01 ± 23.24	NS
DBP (mmHg)	85.20 ± 13.00	84.91 ± 12.61	85.76 ± 13.78	NS
Hypertension no. (%)	215 (83.0)	137 (80.1)	78 (88.6)	NS
Hyperlipidemia no. (%)	72 (28.8)	49 (28.7)	23 (26.1)	NS
Diabetes no. (%)	84 (32.4)	47 (27.5)	37 (42.0)	.018
CAD no. (%)	40 (15.4)	21 (12.3)	19 (21.6)	.050
Smoking no. (%)	111 (42.9)	72 (42.1)	39 (44.3)	NS
Alcohol drinking no. (%)	74 (28.6)	51 (29.8)	23 (26.1)	NS
BMI (kg/m^2^)	23.83 ± 2.99	24.05 ± 2.99	23.41 ± 2.95	NS
Infract volume (cm^3^)	1.32 (0.46–3.70)	1.11 (0.39–2.56)	1.94 (0.77–6.20)	<.001
Hospital stays (days)	9.00 (7.00–12.00)	9.00 (7.00–11.00)	10.00 (8.00–14.00)	.005
NIHSS score on admission, median (IQR)	4.00 (2.00–6.00)	3.00 (2.00– 5.00)	5.00 (3.00–9.00)	<.001
NIHSS score at discharge, median (IQR)	3.00 (1.00–5.00)	2.00 (1.00–4.00)	4.50 (3.00–7.75)	<.001
Laboratory tests				
WBC (109/L)	6.47 (5.50–7.75)	6.53 (5.62–7.66)	6.41 (5.38–8.29)	NS
Neutrophils (109/L)	3.88 (3.15–5.03)	3.83 (3.13–4.63)	4.17 (3.17–5.30)	NS
ALT (IU/L)	19.00 (13.00–27.00)	20.00 (13.00–28.00)	18.00 (14.00–25.00)	NS
AST (IU/L)	22.00 (18.00–26.00)	22.00 (19.00–25.00)	21.00 (17.00–27.75)	NS
LDL‐C (mmol/L)	2.89 ± 0.89	2.86 ± 0.88	2.96 ± 0.91	NS
TC (mmol/L)	4.84 ± 1.10	4.84 ± 1.09	4.85 ± 1.12	NS
TG (mmol/L)	1.57 (1.20–2.19)	1.61 (1.21–2.41)	1.52 (1.13–1.96)	NS
HbA1c (%)	5.90 (5.50–6.83)	5.80 (5.50–6.48)	6.05 (5.58–7.60)	.017
Albumin (g/L)	38.36 ± 3.61	38.72 ± 3.51	37.64 ± 3.73	.022
SCr (μmol/L)	71.00 (60.00–83.00)	72.00 (60.00–84.00)	70.50 (60.00–82.75)	NS
BUN (mmol/L)	4.80 (3.90–5.80)	4.90 (3.90–5.80)	4.50 (3.80–5.70)	NS
Uric acid (mmol/L)	299.56 ± 89.49	308.39 ± 90.62	282.39 ± 85.152	.023
Hs‐CRP (mg/L)	1.82 (0.79–4.28)	1.46 (0.67–3.47)	2.83 (1.11–8.28)	<.001
IL‐33 (ng/ml)	58.76 ± 20.67	62.45 ± 20.50	51.58 ± 19.16	<.001
Location of cerebral infarction no. (%)				
Cerebral lobes	28 (10.8)	21 (12.3)	7 (8.0)	NS
Basal ganglia and Thalamus	118 (45.6)	83 (48.5)	35 (39.8)	
Brainstem and Cerebellum	61 (23.6)	40 (23.4)	21 (23.9)	
Multiple infarction	52 (20.1)	27 (15.8)	25 (28.4)	
Medications, no. (%)				
Antiplatelet agents	184 (84.6)	130 (76.0)	54 (61.4)	.014
Anticoagulation agents	12 (2.6)	6 (3.5)	6 (6.8)	NS
Statins	196 (82.7)	136 (79.5)	60 (68.2)	.044

Abbreviations: ALT, alanine aminotransferase; AST, aspartate transaminase; BMI, body mass index; BUN, blood urea nitrogen; CAD, coronary artery disease; DBP, diastolic blood pressure; HbA1c, glycosylated hemoglobin; Hs‐CRP, high‐sensitivity C‐reactive protein; IL‐33, interleukin‐33; IQR, interquartile range; LDL‐C, low‐density cholesterol; NIHSS, National Institutes of Health Stroke Scale; NS, not significant; SBP, systolic blood pressure; SCr, serum creatinine; TC, total cholesterol; TG, triglycerides; WBC, leukocyte.

### 3.2 Functional outcome

After one year of follow‐up, 88 AIS patients (33.98%) had poorer outcome (BI score ≤ 90). IL‐33 concentrations in AIS patients with good functional outcome were significantly higher than those with unfavorable outcome (62.45 ± 20.50 ng/ml vs. 51.58 ± 19.16 ng/ml, *p* < .001). Basic information on stroke patients with better or poorer outcome is shown in Table 1. According to the univariate logistic regression analysis, IL‐33 level was closely associated with one‐year functional prognosis with an unadjusted OR of 0.973 (95% CI, 0.960–0.987, *p* < .001). After adjustment of confounding confounders whose *p* value were ≤0.2 (age, hypertension, diabetes mellitus, CAD, BMI, cerebral infarct volume, location of cerebral infarction, NIHSS score on admission and at discharge, hospital stays, neutrophils, ALT, TG, Albumin, HbA1c, uric acid, Hs‐CRP, and medications), IL‐33 was still an independent prognostic predictor of AIS with an adjusted OR of 0.956 (95% CI, 0.937–0.976, *p* < .001; Table 2). In addition, NIHSS score on admission and Hs‐CRP were independent factors to predict stroke outcomes.

**Table 2:** Logistic regression model with predictors of unfavorable outcome (*N* = 259)

Characteristics	Unadjusted OR (95% CI)	*p* Value	Adjusted OR (95% CI)	*p* Value
Age (years)	1.029 (1.001–1.057)	.041	—	—
Male no. (%)	0.875 (0.511–1.499)	.627	—	—
SBP (mmHg)	1.005 (0.994–1.017)	.358	—	—
DBP (mmHg)	1.005 (0.985–1.025)	.616	—	—
Hypertension no. (%)	1.936 (0.907–4.131)	.088	—	—
Hyperlipidemia no. (%)	0.881 (0.493–1.573)	.668	—	—
Diabetes no. (%)	1.914 (1.115–3.285)	.019	—	—
CAD no. (%)	1.967 (0.994–3.894)	.052	—	—
Smoking no. (%)	1.094 (0.651–1.838)	.733	—	—
Alcohol drinking no. (%)	0.833 (0.467–1.483)	.534	—	—
BMI (kg/m^2^)	0.929 (0.850–1.015)	.104	—	—
Infract volume (cm^3^)	1.036 (1.003–1.071)	.032	—	—
Location of cerebral infarction no. (%)	1.268 (0.950–1.693)	.107	—	—
Hospital stays (days)	1.111 (1.033–1.196)	.005	—	—
NIHSS score on admission, median (IQR)	1.374 (1.238–1.525)	<.001	1.476 (1.117–1.949)	.006
NIHSS score at discharge, median (IQR)	1.381 (1.236–1.543)	<.001	—	—
Laboratory tests	—	—	—	—
WBC (10^9^/L)	1.083 (0.958–1.225)	.202	—	—
Neutrophils (10^9^/L)	1.160 (1.005–1.338)	.043	—	—
ALT (IU/L)	0.985 (0.964–1.007)	.171	—	—
AST (IU/L)	1.008 (0.985–1.031)	.495	—	—
LDL‐C (mmol/L)	1.137 (0.852–1.517)	.383	—	—
TC (mmol/L)	1.010 (0.799–1.277)	.933	—	—
TG (mmol/L)	0.707 (0.519–0.963)	.028	0.628 (0.400–0.986)	.043
HbA1c (%)	1.200 (1.024–1.407)	.025	—	—
Albumin (g/L)	0.919 (0.855–0.989)	.024	—	—
SCr (μmol/L)	1.001 (0.989–1.013)	.867	—	—
BUN (mmol/L)	1.040 (0.881–1.228)	.642	—	—
Uric acid (mmol/L)	0.997 (0.994–1.000)	.028	—	—
Hs‐CRP (mg/L)	1.072 (1.031–1.114)	<.001	1.052 (1.010–1.096)	.016
IL‐33 (ng/ml)	0.973 (0.960–0.987)	<.001	0.956 (0.937–0.976)	<.001
Medications no. (%)	—	—	—	—
Antiplatelet agents	0.501 (0.288–0.872)	.015	—	—
Anticoagulation agents	2.012 (0.629–6.433)	.238	—	—
Statins	0.551 (0.308–0.987)	.045	—	—

Abbreviations: ALT, alanine aminotransferase; AST, aspartate transaminase; BMI, body mass index; BUN, blood urea nitrogen; CAD, coronary artery disease; CI, confidence interval; DBP, diastolic blood pressure; HbA1c, glycosylated hemoglobin; Hs‐CRP, high‐sensitivity C‐reactive protein; IL‐33, interleukin‐33; IQR, interquartile range; LDL‐C, low‐density cholesterol; NIHSS, National Institutes of Health Stroke Scale; R, odds ratio; SBP, systolic blood pressure; SCr, serum creatinine; TC, total cholesterol; TG, triglycerides; WBC, leukocyte.

**Figure 1:**
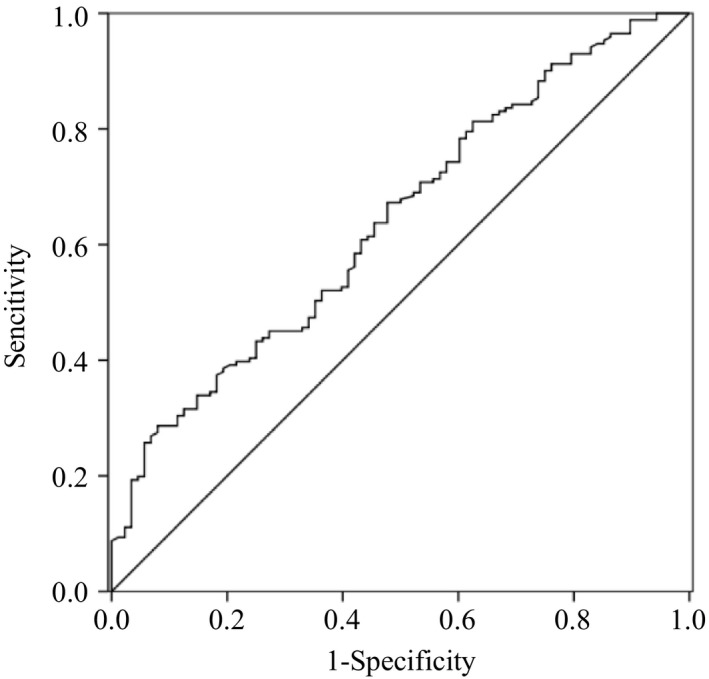
The ROC curve of IL‐33 to predict one‐year functional outcome

**Figure 2:**
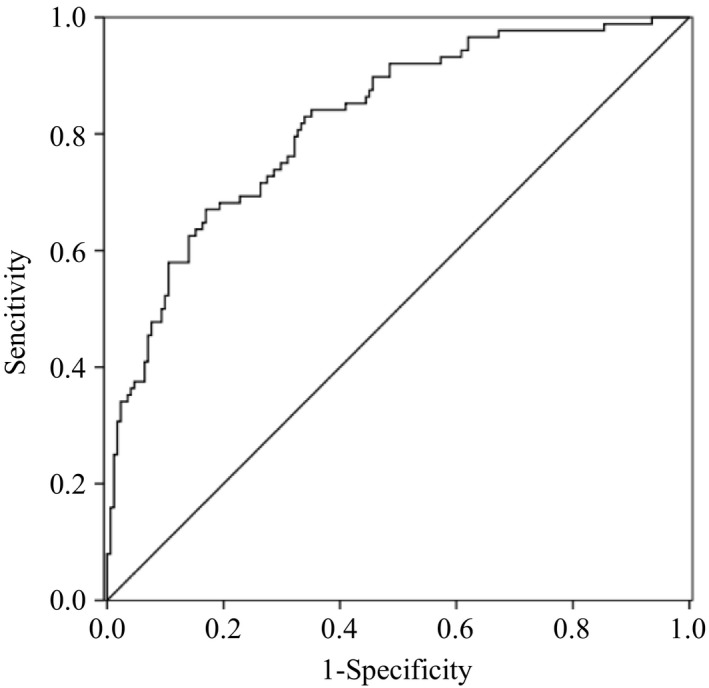
The combinational ROC curve of IL‐33 and NHISS to predict long‐term outcome

ROC curves presented predictive accuracies for IL‐33 levels with (AUC: 0.640, 95% CI: 0.579–0.709, *p* < .001; Figure 1) and the NIHSS score on admission (AUC: 0.722, 95% CI: 0.655–0.789, *p* < .001). The combination of IL‐33 levels and NIHSS score on admission showed a higher predictive accuracy (AUC: 0.822, 95% CI: 0.769–0.875, *p* < .001; Figure 2) than the IL‐33 levels or NIHSS score alone.

### 3.3 Recurrent ischemic stroke

Over 2.17 ± 0.57 years of follow‐up, 35 patients (13.5%; average age of 66.44 ± 8.39 years) were confirmed to have relapsed episode of ischemic stroke, while 45 patients were unreachable for questioning about their health status. Serum IL‐33 level was significantly lower in patients with recurrent ischemic stroke as compared to those without recurrence (50.68 ± 17.26 ng/ml vs. 60.06 ± 20.91 ng/ml, *p* = .011]. In Cox regression analysis, IL‐33 level was associated with recurrent ischemic stroke with an unadjusted HR 0.980 (95% CI 0.964–0.996, *p* = .016). After adjustment for potential confounders whose unadjusted *p* values were ≤0.2 in Cox regression analysis (age, gender, CAD, current smoking, diastolic blood pressure, BMI, NIHSS score on admission, the WBCs, LDL‐C, TC), IL‐33 level was still closely related to recurrent episode of ischemic stroke with an adjusted HR 0.979 (95% CI 0.979–0.997, *p* = .025). Based on the ROC curve, IL‐33 showed a significantly great ability to predict ischemic stroke recurrence with an AUC of 0.626 (95% CI, 0.537–0.714, *p* = .016).

## 4 DISCUSSION

This study is the first in our opinion to match IL‐33 levels to long‐term outcomes and recurrence of ischemic stroke in patients after AIS. It turned out that a lower IL‐33 levels are associated with one‐year unfavorable outcome and recurrent ischemic stroke in AIS patients. Moreover, IL‐33 may be used as a prognostic biomarker for long‐term prognosis and recurrence after AIS.

Inflammatory cascade has been implicated in the ischemic processes at different stages of stroke. Responsive mechanisms after stroke could either result in further injury to the brain or may contribute to repair processes (McCombe & Read, 2008). During acute cerebrovascular accidents, the integrity of blood–brain barrier gets compromised which allows for infiltration of peripheral T cells and their subsets to the region of cerebral injury (Gelderblom et al., 2009). Activated T cells could be roughly classified into type 1 and the type 2 activation (Hu et al., 2012). Th1‐type response plays a dominant role in the early stages of AIS through the induction of macrophage polarization into M1‐type which produces pro‐inflammatory cytokines and neurotoxic substances, thereby aggravating secondary ischemic damage (Denes, Humphreys, Lane, Grencis, & Rothwell, 2010; Kigerl et al., 2009). Th2‐type response, however, induces macrophage polarization into M2‐type and therefore promotes the secretion of anti‐inflammatory cytokines, like interleukin‐4 (IL‐4; Butovsky et al., 2006). Th2‐type cells could therefore override and suppress the cell destructive response by Th1‐type response and promote regenerative processes (Schroeter & Jander, 2005). It is therefore not surprising that the balance of Th1/Th2 type response is hypothesized as a clue to regulate inflammatory response.

IL‐33, identified as a novel member of the IL‐1 cytokine family, enhanced the effect of Th2‐type response through the interaction with its heterodimeric receptor ST2 and the IL‐1 receptor accessory protein (Chackerian et al., 2007). IL‐33 could also increase the concentrations of anti‐inflammatory mediators (such as interleukin‐4, interleukin‐5, and interleukin‐10) in serum, and at the same time reduce the concentrations of pro‐inflammatory cytokines (interferon‐γ and interleukin‐17; Jiang et al., 2012; Pomeshchik et al., 2015). By tilting the Th1/Th2 balance toward Th2 cells, IL‐33 performed a protective function in various diseases. In 2008, Miller et al. (2008) suggested that IL‐33 could delay the progression of atherosclerosis. Further studies, Miller and his team in 2015 (Miller et al., 2010) reported that IL‐33 might perform a novel atheroprotective function in adipose tissue inflammation and treatment with IL‐33 could induce beneficial effects on glucose homeostasis and body composition. In addition, Rui et al. (2012) found that decreased concentrations of IL‐33 would exaggerate myocardial ischemia reperfusion injury in mice with diabetes mellitus. Moreover, a recent study has showed that IL‐33 was highly expressed in the spinal cord and brain and played critical roles in central nervous system diseases (Han, Mi, & Wang, 2011). Furthermore, it was demonstrated that genetic variants in IL‐33 gene might be associated with the decreased risk of Alzheimer disease (Chapuis et al., 2009). Fu et al. (2016) implied that IL‐33 played a neuroprotective role and was a potential therapeutic approach for Alzheimer disease. Conversely, another recent study has suggested that IL‐33 might induce glial cells to release inflammatory molecule and exacerbate neuroinflammation in Alzheimer disease (Xiong et al., 2014).

With regard to AIS, the function of IL‐33 in cerebral ischemic infarction was still ambiguous. One recent research demonstrated that the genetic variation in IL‐33 was significantly related to risk of ischemic stroke in north Chinese population (Guo, Zhou, Guo, Zhang, & Sun, 2013). Luo et al. (2015) found that IL‐33 has protective effects after middle cerebral artery occlusion and could be used as a novel therapeutic target for treatment of ischemic stroke. Subsequently, Korhonen et al. (2015) carried out a series of experiments in vivo and in vitro concluding that IL‐33 might represent a novel therapeutic approach of stroke. In addition, recent study revealed that IL‐33/ST2 could serve as an immune regulatory mechanism which mitigated brain damage after AIS (Yang et al., 2017). Furthermore, a cohort study conducted by Li et al. (2016) showed that higher IL‐33 level was related to smaller infarction volume and lesser stroke severity, and with increased serum IL‐33 levels patients presenting with more favorable functional outcome at 3 months. However, in another contrasting research, elevated levels of IL‐33 correlated with larger infarction volume due to the regulation of inflammatory reaction by IL‐33 (Liu, Xing, Gao, & Zhou, 2014). In this study, we hypothesize that IL‐33 might be beneficial to the reparative process of AIS since IL‐33 levels were increased in patients with good prognosis.

Our study also identified several limitations which should not be ignored. Firstly, all patients were recruited from one hospital which might result in selection bias. Secondly, the lack of research objects and the low incidence of recurrent ischemic stroke caused insufficient power for IL‐33 levels to predict stroke recurrence. Finally, serum IL‐33 levels were assessed only once at admission; thus, further studies should explore how circulating IL‐33 levels dynamically changes after AIS.

## 5 CONCLUSION

In this study, we found that lower serum IL‐33 levels were associated with worse prognosis and recurrence of first‐ever stroke. Therefore, the measurement of IL‐33 level might be considered as a predictive factor for the long‐term outcomes and ischemic stroke recurrence. However, further researches are required to verify our result.
